# Understanding Charge Dynamics in Dense Electronic Manifolds in Complex Environments

**DOI:** 10.1021/acs.jctc.2c00794

**Published:** 2023-01-05

**Authors:** Fulvio Perrella, Alessio Petrone, Nadia Rega

**Affiliations:** † Department of Chemical Sciences, University of Napoli Federico II, Complesso Universitario di M.S. Angelo, via Cintia 21, I-80126, Napoli, Italy; ‡ Scuola Superiore Meridionale, Largo San Marcellino 10, I-80138, Napoli, Italy; ¶ Istituto Nazionale Di Fisica Nucleare, sezione di Napoli, Complesso Universitario di Monte S. Angelo ed. 6, via Cintia, 80126, Napoli, Italia; § CRIB, Centro Interdipartimentale di Ricerca sui Biomateriali, Piazzale Tecchio 80, I-80125, Napoli, Italy

## Abstract

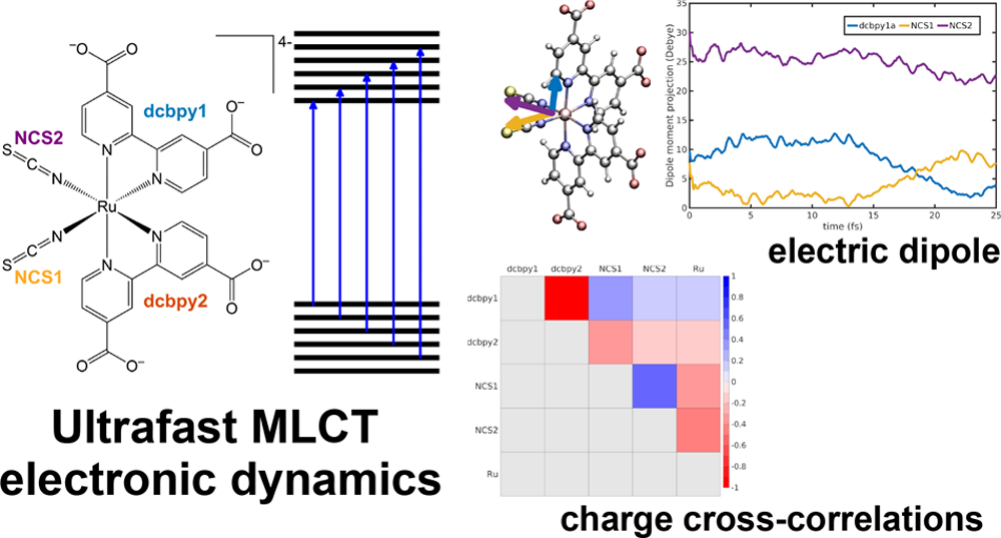

Photoinduced charge transfer (CT) excited states and their relaxation mechanisms can be highly interdependent on the environment effects and the consequent changes in the electronic density. Providing a molecular interpretation of the ultrafast (subpicosecond) interplay between initial photoexcited states in such dense electronic manifolds in condensed phase is crucial for improving and understanding such phenomena. Real-time time-dependent density functional theory is here the method of choice to observe the charge density, explicitly propagated in an ultrafast time domain, along with all time-dependent properties that can be easily extracted from it. A designed protocol of analysis for real-time electronic dynamics to be applied to time evolving electronic density related properties to characterize both in time and in space CT dynamics in complex systems is here introduced and validated, proposing easy to be read cross-correlation maps. As case studies to test such tools, we present the photoinduced charge-transfer electronic dynamics of 5-benzyluracil, a mimic of nucleic acid/protein interactions, and the metal-to-ligand charge-transfer electronic dynamics in water solution of [Ru(dcbpy)_2_(NCS)_2_]^4–^, dcbpy = (4,4′-dicarboxy-2,2′-bipyridine), or “N3^4–^”, a dye sensitizer for solar cells. Electrostatic and explicit ab initio treatment of solvent molecules have been compared in the latter case, revealing the importance of the accurate modeling of mutual solute–solvent polarization on CT kinetics. We observed that explicit quantum mechanical treatment of solvent slowed down the charge carriers mobilities with respect to the gas-phase. When all water molecules were modeled instead as simpler embedded point charges, the electronic dynamics appeared enhanced, with a reduced hole–electron distance and higher mean velocities due to the close fixed charges and an artificially increased polarization effect. Such analysis tools and the presented case studies can help to unveil the influence of the electronic manifold, as well as of the finite temperature-induced structural distortions and the environment on the ultrafast charge motions.

## 1 Introduction

Photoinduced charge transfer (CT), defined here as the physical phenomena where electrons (or a fraction of an electronic charge) transfer between states or between regions of space of the system,^1^ has a pivotal role in photosynthesis, biochemistry, and technological applications, such as generation and storage of electricity, photovoltaic cells, organic chromophores (i.e., light-emission diodes).^2−6^ Thus, providing a molecular interpretation of the ultrafast (subpicosecond) interplay among initial photoexcited states is crucial for improving and understanding such phenomena.^7,8^ Among nonperturbative approaches to mean-field quantum electronic dynamics (ED), real-time time-dependent density functional theory (RT-TDDFT) has been proven to be very powerful in these regards, since via real-time methods we can explicitly propagate in time the electronic density by evolving the time-dependent Schrödinger equation.^9,10^ In particular, in this paper we focus on the large potential of RT-TDDFT in describing, simulating, and interpreting on the molecular scale the ultrafast charge recombination, and more in general photoinduced charge dynamics.

From a more general perspective, TDDFT, even in its linear response formalism with standard approximate functionals, can have issues in modeling some excited states, including those with charge-transfer, Rydberg, or double excitation characters. Wave function-based methods can partially overcome this issue,^11^ although real-time methods built upon the configuration interaction expansion of the wave function can suffer from truncation effects. Recently, coupled-cluster real-time based approaches have shown to have large potentiality,^12−14^ although this methodology can become soon computationally prohibitive for systems of large size and in condensed phase. On the other hand, an accurate choice and calibration of the density functional can improve the accuracy of the treatment of charge-transfer excitations. As matter of fact, RT-TDDFT has been proven capable to directly and accurately model the charge-transfer and exciton dynamics in several donor–acceptor systems,^15−20^ providing a molecular interpretation of the interplay between initial photoexcited states,^15,21−27^ exciton and polaron formation,^17,28−31^ including also relativistic effects.^32,33^ We refer readers to previous review publications^9,10^ for more detailed discussion on the subject.

Photoinduced CT processes can be highly interdependent on the environment effects, such as solvent, biological, or polymeric matrices. The excited state relaxation mechanism can be influenced by several weak interactions (i.e., solute–solvent) and the consequent changes in the electronic density. A more detailed description of the system, including the surrounding environment in explicit way with more accurate methods, cannot be avoided. This is a huge challenge for the theoretical study of the interplay between molecules in complex environments (i.e., solutions) and their related nonequilibrium properties. The molecules surrounding the system under investigation can highly influence the resulting CT and the consequent electron mobility in condensed phase.^34−41^ Hybrid quantum/classical (QM/MM) methods and more general multilayer computational schemes have been proven very useful to probe and characterize the photoinduced dynamics of macromolecular systems, even large biomolecules in complex environments.^42−53^ The most common examples of hybrid QM/MM models introduce the effect of the environment on the QM part with an atomistic charge distribution to mimic the surrounding molecules. This electrostatic embedding approach has been shown to capture the large part of the environment effects, but it can fail in describing the mutual polarization between the QM and the MM parts. To overcome this issue, polarizable MM force fields have been proposed,^54−65^ and have recently used in combination with RT-TDDFT for optical spectra.^66^ On the other hand, the way of equilibrating the QM and MM parts can be problematic if the charge dynamics needs to be followed on the electronic time-scale; thus, in this work we analyze the effects on the CT mechanisms and kinetics of explicitly introducing a large portion of the environment at QM level.

From a methodology perspective, once time-dependent electronic density is collected, electric dipoles and molecular orbital occupation numbers are monitored along the RT-TDDFT trajectories for CT analysis. These quantities can satisfactorily describe both the time and spatial evolution of the electronic density only for systems of limited size characterized by photoinduced excitations involving well separated electronic states with few molecular orbital contributions. Here, we focus on the development of a suite of analysis tools for providing a direct molecular picture of charge transfer, when intricate charge dynamics are in play involving also dense electronic manifolds.

**Figure 1:**
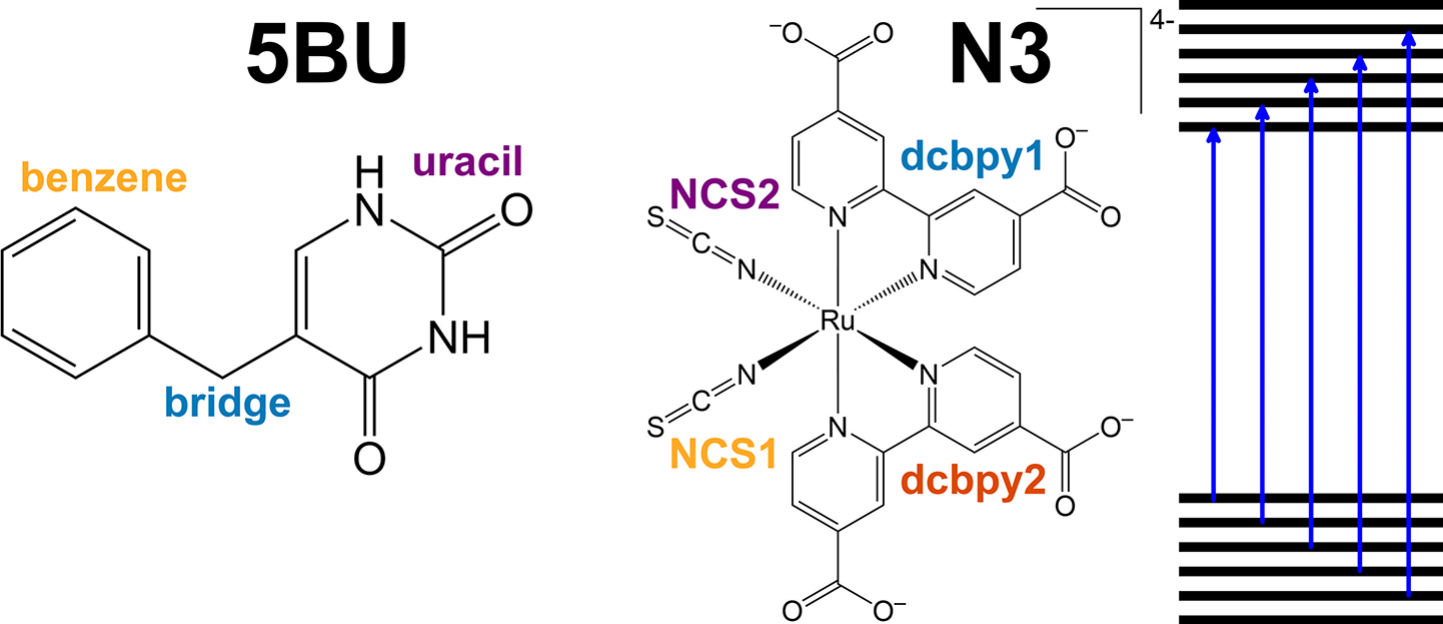
Molecular structures of the model systems employed for electronic dynamics analysis protocol. Left: 5-benzyluracil (5BU). Right: [Ru(dcbpy)_2_(NCS)_2_]^4–^ (N3^4–^) and a schematic and pictorial layout of the closely energy spaced MOs in a transition metal complex. Arrows represent one particle excitations in terms of MOs involved in the photoinduced transitions creating the dense manifold.

5-Benzyluracil (5BU, please refer to Figure 1, left panel, for its structure)^67^ is a first and smaller CT system with respect to metal complexes that still presents a nontrivial electronic dynamics, which was chosen as an optimal proof of concept model to test such protocol, based upon easy to be read cross-correlation maps to highlight CT phenomena. 5BU is a model of the photoinduced interactions between nucleic acids nucleobases and proteins aromatic residues, which can lead to a cross-linking reaction. The central CH_2_ bridge allows to keep the two moieties close, as in a real nucleic acid/protein complex. 5BU relaxation mechanisms have been already investigated in several experimental time-resolved spectroscopy studies.^68−71^ Besides uracil and benzene-localized excitations, 5BU features a higher-energy B → U CT state, which could be implied in the observed photochemistry.

On the other hand, transition metal complex metal-to-ligand charge-transfer (MLCT) excited states have gained a paramount importance, given also these photoactivated species are widely used for light harvesting and photocatalysis.^72−76^ Recently, their range of applications have expanded in the field of circular economy and green chemistry, given their importance as alternative approaches to solar activation of nanostructured systems widely employed in the biomass conversion to solar fuels and the selective production of fine chemicals from agricultural residues and food processing waste.^77,78^ Among the most studied and employed nanostructured systems in this field, there are the dye-sensitizers for TiO_2_, mostly represented by the Ru-based polypyridyl complexes. In this regard, [Ru(dcbpy)_2_(NCS)_2_]^4–^, dcbpy = (4,4′-dicarboxy-2,2′-bipyridine), or “N3^4–^” (please see Figure 1), here used as a second, more challenging, test system, belongs to a broad class of transition-metal compounds undergoing rapid and complex CT dynamics, strongly correlated with both a structural rearrangement and the polar environment (i.e., solvent).^73,79−88^ N3, and its charged variants, is characterized by a complex dynamics that can involve multiple electronic states from the singlet initial ^1^MLCT photoinduced state(s) to the long-lived final triplet ^3^MLCT both in solution^89−94^ and on semiconductor substrates.^72,95−101^ This suggests that in N3 and related Ru compounds, many electronic states in the excited ^1^MLCT manifold are involved and each one can potentially lead to different relaxation pathways, as pointed out by several theoretical studies, employing nonadiabatic molecular dynamics, also including spin–orbit couplings.^102−113^ For this system, moreover, the electronic relaxation pathway involves a concurrent intramolecular vibrational energy redistribution and solvation relaxation^114−117^ and the localization (or not) of the photoexcited electron on one (or more) acceptor ligand(s) may be crucial to the occurrence and the mechanism of CT processes.

RT-TDDFT is here the method of choice to observe the charge density, explicitly propagated in the ultrafast time domain, along with all time-dependent properties that can be easily extracted from it. In such short simulated time, it could be assumed that the effect of nuclear vibrations is still limited. Therefore, the “fixed nuclei” approximation implied in these purely electronic dynamics is reasonable. Moreover, for N3^4–^ model system, the electronic density can be propagated among only the singlet manifold if the ultrafast simulated time is below the intersystem crossing (ISC) time-scale, which, for N3 and [Ru(bpy)_3_]^2+^, is, on average, experimentally estimated above 30 fs.^118−126^ Of course, longer simulations should require vibronic and spin–orbit couplings to allow full relaxation and singlet–triplet ISC, as recognized in the literature.^102,104,105^

In this work, we introduce a designed protocol of analysis for real-time ED to be applied to time evolving electronic density related properties to characterize both in time and in space CT dynamics in complex systems. As case studies, we present the employment of such protocol to the 5BU B → U CT and N3^4–1^MLCT photoinduced dynamics involving in the latter case a dense manifold of electronic states, proposing easy to be read cross-correlation maps to detect CT phenomena. These suite of analysis tools to be combined with RT-TDDFT dynamics, performed including large portion of the environment at QM level and describing the resulting part of the system as point charges, provided direct access to ultrafast charge reorganizations in complex electronic manifolds. This can represent a very useful insight, since CT can not be easily experimentally probed even using the most recent experimental time-resolved spectroscopic techniques, given the subpicosecond nature of the ultrafast charge dynamics processes.

## 2 Methodology

To test our methodology, we performed RT-TDDFT ED simulations of 5BU and N3^4–^ model systems to shed light on the CT dynamics following the photoexcitation. The CT state chosen for 5BU is located in the UV region at ∼49700 cm^–1^, above the B and U localized π → π* and *n* → π* states (Table S1), and it has a distinct B → U character, as suggested by Natural Transition Orbitals (NTOs) and hole–electron correlation plots from transition density population analysis (Figure S5).^127,128^ The simulated N3^4–1^MLCT state is experimentally found instead around 25000–25300 cm^–1^ (400–395 nm), still in the visible range, and it is relevant for 2D spectroscopy experiments and light harvesting performances.^129^ Such ^1^MLCT state was modeled in the initial photoexcitation of the molecule as a sudden change in the charge density localized on metal toward the dcbpy acceptor ligands (please refer to Figure S6 in the ESI for a characterization of ^1^MLCT state through Natural Transition Orbitals (NTO), Figure S7 for hole–electron correlation plots and to Table S2 for calculated energies and MO main contributions). In this way, we ensure to realistically mimic the Franck–Condon excitation of the 5BU and N3^4–^ molecules occurring in the experiment and the resulting electronic density dynamics was then tracked in space and time. For N3^4–^ in particular, the CT dynamics is influenced by the dense electronic manifold that arises from the small energy separation among the valence molecular orbitals (MOs) and the virtual MOs as well (see Figure 1 for a schematic representation of N3^4–^ MOs layout).

We first present a usual MO occupation number dynamics. Such analysis usually can help in understanding the spatial evolution of charge, although in systems with dense and convoluted MO layouts, such as in metal complexes, it is not very useful. Then, other important features such as the electric dipole moment and its frequency response are also computed to provide a complete description of the mechanism and kinetics of the electronic dynamics. Finally, CT dynamics is highlighted by introducing easy to be read cross-correlation maps (see following discussion).

Such analysis tools can help moreover to unveil the influence of finite temperature and environment effects on the ultrafast charge motions. Explicit solvation effects onto the CT dynamics were modeled in N3^4–^ by including the surrounding water molecules. Solvent polarization effects were included as water molecules embedded atomic charges (as usual electrostatic embedding); then a larger portion of water molecules was treated at *ab initio* level, and only the more distant ones were left as atomic charges (see Simulation Details section). The suite of proposed analysis tools is thus exploited to highlight the effects of water polarization on N3^4–^ CT kinetics and mechanism.

### 2.1 Simulation of the CT Dynamics Using RT-TDDFT

The ultrafast electronic dynamics of representative 5BU CT and N3^4–1^MLCT photoinduced charge-transfer excited states were characterized and propagated, taking into account for the N3^4–^ simulations also the finite temperature effects on the molecular structure and solvation effects on the dynamics (see Simulation Details subsection).

Electronic dynamics were performed on fixed nuclear configurations, since we were focused on the ultrafast electronic reorganization after CT events. In these early stages of dynamics (∼25 fs), we can neglect the nuclear motion influence on the charge reorganization. As concerning the initial system geometries, the optimized minimum energy geometry (5BU) or sets of representative nuclear configurations from water solution *ab initio* molecular dynamics (N3^4–^) were selected (see Simulation Details subsection).

As previously mentioned, this computational experiment can provide the explicit evolution of the electronic density in time, mathematically represented in terms of time evolving orbital coefficients or, as in this study, of the one-electron density matrix **P** in an orthonormal atom centered basis. The time evolution of a specified initial photoexcitation is resolved through RT-TDDFT calculations, in which the electronic density matrix is propagated in time according to the nonlinear Liouville–von Neumann equation^130,131^
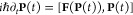
1where *i* is the imaginary unit, *ℏ* is the reduced Planck constant, ∂_*t*_ is the partial derivative with respect to the time and **F** is the Fock (Kohn–Sham within DFT framework) matrix in the orthonormal basis, including in this work also the polarization by the environment. Formally, eq 1 may be solved (propagated) exactly in the time domain (given an initial time *t*_0_) through the Magnus expansion of the time-domain propagator^132^
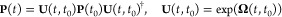
2where the † symbol is the Hermitian conjugate and **Ω**(*t*, *t*_0_) is a nonterminating series expansion which must be truncated in practice. We used a modified development version of the Gaussian electronic structure software package,^133^ and as integration scheme we adopted the modified midpoint unitary transformation (MMUT) method of Li et al.^24^ MMUT is a symplectic multistep (leapfrog) explicit integration scheme based on the Magnus expansion with error formally Δ*t*^2^ (*t*_*k*_ is the current time step during the time propagation)
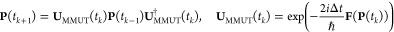
3

The easiest procedure for preparing an initial state resembling an electronic excitation is to directly adjust the orbital populations without relaxation by promoting an electron to an unoccupied orbital,^28^ although more specific and experimental-tailored approaches have been proposed over the years.^134−137^ Thus, in order to obtain the initial electronic perturbation to perform electronic dynamics, the excited states of interest are prepared by promoting an electron from a selected occupied molecular orbital to one that is unoccupied in the ground state (“Koopman excitation”) according to the electronic transition of interest between the singlet ground state (S_0_) and the n-th singlet excited state (S_*n*_), whose main orbital contributions are resolved using preliminary frequency domain linear-response (hereafter LR)-TDDFT calculations. The “Koopman excitation” step creates a nonstationary electron density that is representative of a coherent superposition of the ground and excited states of interest, according to a well-established procedure.^9,17,31,138−140^

Comparison of spatial and electric dipole features allowed us to associate the S_*n*_ states involved in the S_0_ → S_*n*_ transitions with the ^1^MLCT state, responsible for the ultrafast dynamics observed experimentally in the 2D electronic-vibrational spectra.^129^

### 2.2 Tools for Analyzing Space and Time CT Dynamics

To disentangle the most relevant features underlying the electronic dynamics of the chosen model systems CT states, several parameters were evaluated along the RT-TDDFT trajectories and analyzed both in time and frequency domains. Time-dependent properties were extracted for this aim from the time evolving density.

First, to try to provide a spatial representation of the CT dynamics, the occupation of molecular orbitals, *n*_*i*_(*t*), was followed in time:
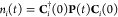
4where the time-evolving electronic density matrix is projected onto the ground state molecular orbitals (MO) basis (represented as a linear combination of atomic orbital basis at time zero, ***C***(0)) and *n*_*i*_(*t*) is the occupation number of the  ground state MO. In particular, the occupation of several frontier orbitals (from HOMO–20 to LUMO+20) was tracked.

Then, the time-dependent electric dipole moment **μ**(*t*) was computed, since it is a very useful quantity to characterize the evolution of charge transfer states. This quantity can be calculated as
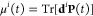
5where ***d*^*i*^** (*i* = *x*, *y*, *z*) is the *i*-component of the one-electron electric dipole moment operator in the atomic orbital basis ({ϕ_α_}), , and Tr is the trace operator.

### 2.3 Cross-Correlation Maps in CT Dynamics

In this work, we present for the first time a suite of tools to visualize and interpret in an intuitive way the ultrafast (femtosecond) photoinduced CT dynamics. In this regard, electronic density usually can be partitioned and then analyzed in terms of charges. Different spatial regions of the system can be used to group the charges according to different fragments.

**Figure 2:**
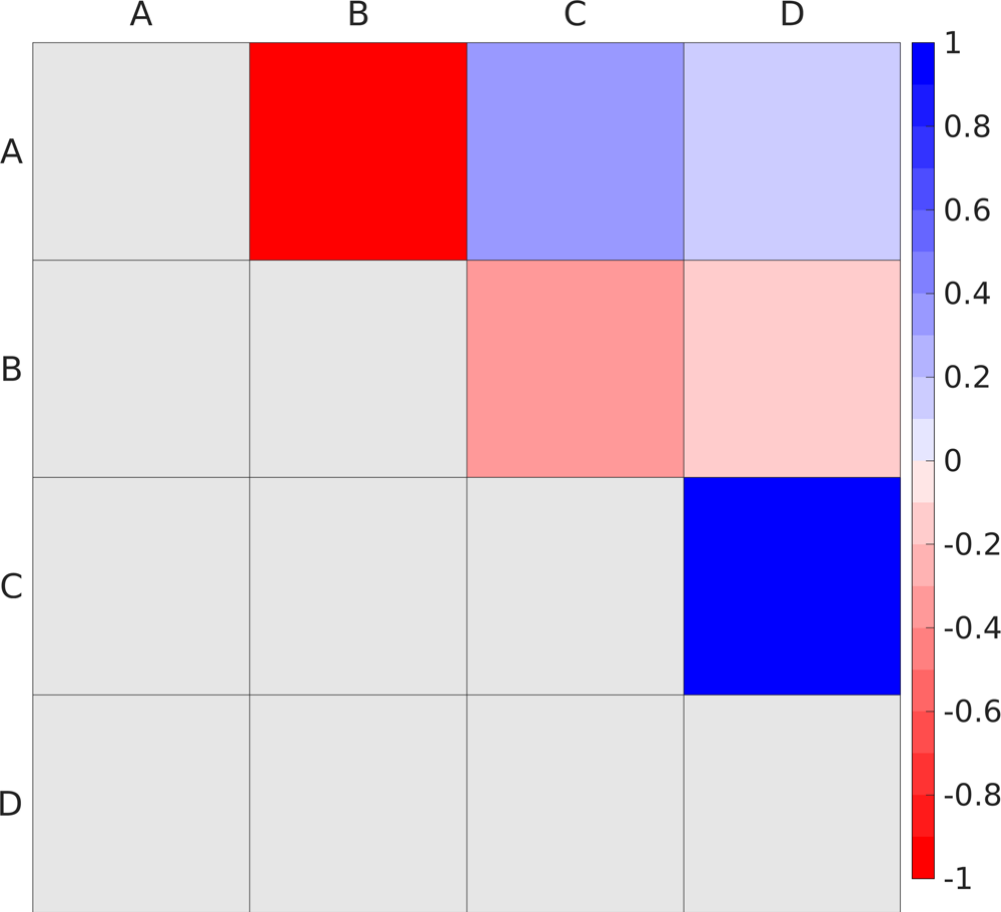
Example of a charge cross-correlation map. The molecular fragments are called A, B, C, and D. Maximum *R*_*xy*_ is reported for each unique x/y pair (A/B, A/C, A/D, B/C, B/D, C/D) through a red-blue color scale. So, for instance, the C/D and A/B pairs show a high positive or negative correlation, respectively. Only the upper triangle is shown, since A/B and B/A cross-correlations are equal and the A/A autocorrelations are equal to 1.

The 5BU model system has two distinct benzene and uracil moieties, linked by a CH_2_ bridge. Each of them can be therefore considered as a distinct fragment for time-dependent density partition (Figure 1, left panel). On the other hand, the N3^4–^ system belongs to *C*_2_ point group (if not distorted); thus, the molecule has been partitioned into five fragments, comprising the two dcbpy ligands, the two NCS^–^ ligands, and the Ru(II) metal ion (Figure 1, right panel). Actually, for the N3^4–^ optimized structures NCS1 and NCS2, dcbpy1 and dcbpy2 are related by symmetry. Time-evolving group charge differences with respect to the equilibrium ground state ones are obtained by atomic charges summation over each molecular fragment. Since charge dynamics can be not easy to understand, we propose fragment charge cross-correlations and easy to be read maps to aggregate the results. For this aim, we present here the application of time signal analysis tools, such as cross-correlation functions, to time evolving electronic density related properties to characterize CT phenomena. More in details, here we employ a tailored cross-correlation analysis both in time and frequency domains to offer a direct and easy to be read interpretation of the charge density transfer and redistribution mechanisms following the initial photoexcitation, also in water solution. Consider *x*(*t*) and *y*(*t*) as two distinct (average-subtracted) time-series (such as time-dependent group charges); their normalized cross-correlation at time-lag τ was evaluated as
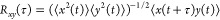
6where the average (denoted as ⟨·⟩) was taken over the length of the electronic dynamics and the  normalization factor allows *R*_*xy*_(τ) to assume values in the [−1, 1] interval. A systematic evaluation of the cross-correlation for each unique pair of time-dependent group charges can be collected into a cross-correlation map such as that shown in Figure 2. A high positive (in phase) correlation or a high negative anti correlation (out of phase) between different spatial regions (i.e., fragments) appears as bright blue or red squares, respectively.

Besides time domain analysis, it is also useful to use the frequency components of the correlation among different dynamical quantities. Thus, here we propose to also analyze the cross power spectrum *S*_*xy*_(ν) (i.e., the Fourier transform of the cross-correlation function) that allows to detect common frequencies shared by *x*(*t*) and *y*(*t*). If calculated from time-dependent group charges, the frequency ruling the charge reorganization between two distinct molecular fragments (and so the charge carriers mobility) can be estimated. The range of frequencies of the cross power spectrum can highlight charge dynamics that can potentially couple with nuclear motions (spectral region under 4000 cm^–1^) and/or electronic states (region above ∼4000 cm^–1^).

### 2.4 Simulation Details

All calculations were performed using the development version of the Gaussian suite of programs.^133^ The electronic structures were obtained by solving the Kohn–Sham equation using for the 5BU model system the long-range-corrected, global hybrid Becke, 3-parameter, Lee–Yang–Parr density functional^141^ (CAM-B3LYP) with the 6-31+G(d,p) basis set, while for the N3^4–^ one, the B3LYP functional^142−144^ with the def2-SVP^145^ basis set and associated electronic core potential (ECP) for Ru.^146^ These levels of theory were already validated in previous studies of respective systems.^69,129^ Regarding the different choice of the DFT functional, range-corrected and range-separated hybrid functionals employ a different treatment of exchange-correlation that can potentially improve the description of charge-transfer states resolved by linear response TDDFT, but it is unclear whether the advantages of their spatially inhomogeneous treatment of exchange energy will faithfully translate from frequency domain response type calculations to the real-time propagation. Additionally, the role of range separated hybrid functionals (i.e., CAM-B3LYP) has also been shown to be not mandatory for the accurate description of the electronic excitations in the pumping region for Ru compounds similar to N3^4–^.^147^ Finally, the level of theory employed for N3^4–^ has been proven to be accurate for the structural characterization of both the S_0_ and T states in water solution along with their related X-ray transitions.^34,148,149^

All the electronic dynamics trajectories were simulated for 25 fs. A time step of 1 as (5BU and N3^4–^ D-G and D-C structures, see following discussion) or 2 as (N3^4–^ D-W structure) allowed an energy conservation within 10^–6^ hartree.

The N3^4–^ model system was also employed to study the explicit distortion effects by vibrations and the polar environment on the ultrafast charge dynamics in water solution. In particular, a representative snapshot of the average structural and solvation arrangement of the system at room temperature was extracted from a previously collected ground state *ab initio* molecular dynamics (AIMD) of N3^4–^ in explicit water solution, according to the procedure explained in refs (34),^149^. In brief, the energy potential ruling the AIMD was constructed according to the scheme described in refs (34, 150−152). N3^4–^ in water solution was modeled by a spherical box (of 22 Å radius) comprising the N3^4–^ molecule, treated at a DFT level, and 1462 water molecules at an MM level (to include at atomistic level at least three solvation shells around the ligands), described by the TIP3P force field. For interested readers, please refer to ref (149) and to Section 1 in the ESI for AIMD details.

**Figure 3:**
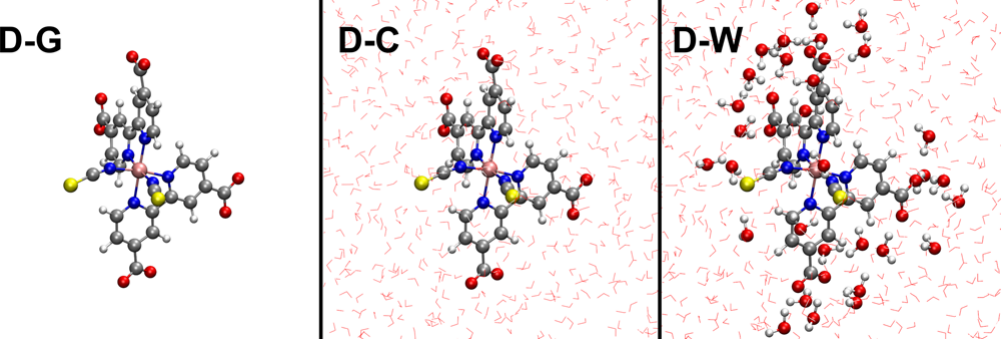
N3^4–^ structures employed in ^1^MLCT excited state propagation. D-G: MD snapshot, only N3^4–^; D-C: MD snapshot, N3^4–^ + water atomic point charges; D-W: MD snapshot, N3^4–^ + first-shell DFT water + water charges.

This low-symmetry N3^4–^ structure extracted from MD (reported in Figure 3) can account for vibrations and both indirect and direct solvent effects on ultrafast charge reorganization in the photoinduced CT states. It should be remarked that the aim is to observe how the deviation from the ideal symmetry, due to the previously cited factors, affects the purely electronic dynamics in a “fixed nuclei” approximation, which is reasonable in the simulated time-scale, and not to include vibronic couplings, required for a nonadiabatic molecular dynamics. Starting from the gas-phase N3^4–^ structure from MD (D-G structure, see Figure 3 for labels), explicit solvation effects onto the excited states electronic dynamics were added to N3^4–^ by explicitly including the surrounding water molecules. As a first approximation, solvent polarization effects were included as water molecules embedded atomic charges (D-C structure, Figure 3). Then, to evaluate and correct the overpolarization effects due to the fixed point charges used in the electrostatic embedding scheme, the first-shell water molecules were treated at *ab initio* level, and only the more distant ones were left as atomic charges (namely D-W structure in Figure 3).

Mulliken population analysis^153,154^ was employed to obtain time-dependent fragment charges along RT-TDDFT trajectories. Although this choice is due to the current implementation of real time dynamics in the development version of the code, the dependence of the results upon this analysis is mitigated by summing the obtained atomic charges over molecular fragments. In order to test the effects of choosing Mulliken analysis over other options, fragments charge differences with respect to ground state at *t* = 0 (i.e., at the beginning of the excited state propagation) were computed also with Natural Population Analysis (NPA) approach^155^ (see Tables S7 and S8 in the ESI for a comparison between Mulliken and NPA charges). In particular, maximum deviations about only 0.050 *e* for 5BU and 0.028 *e* for N3^4–^ of Mulliken relative group charges from the NPA ones can be estimated.

## 3 Results and Discussion

### 3.1 5BU Model System

#### 3.1.1 Electronic Dynamics Characterization through MO Occupation Numbers

**Figure 4:**
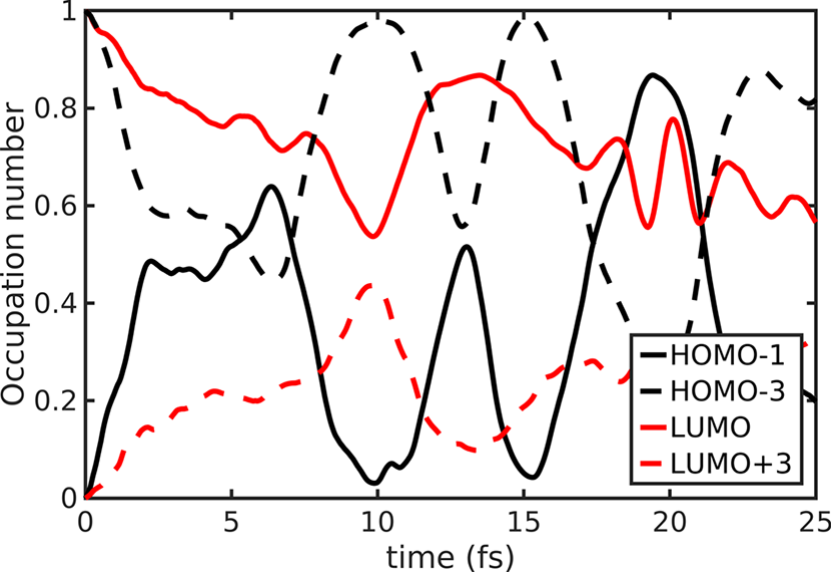
MOs occupation number dynamics in 5BU CT excited state propagation.

The ultrafast evolution of the photoexcited 5BU CT state is first described through frontier MOs occupation dynamics. Partial (up to 0.5 e^–^) oscillations seem to affect both the hole and the electron. In particular, the hole (initially photogenerated on the benzene-localized HOMO–1) is partially exchanged with the inner, uracil-localized, HOMO–3. At the same time, the electron photogenerated in the uracil-centered 5BU LUMO is partially shared with the LUMO+3, localized on the benzene moiety (Figure 4). The ultrafast dynamical picture of the first 5BU B → U CT state offered by the MO occupations therefore suggests a possible hole/electron recombination. In fact, both move toward the central CH_2_ bridge and so they can get closer, in principle, after the initial photoexcitation, although their oscillation actually appears quite uncorrelated.

#### 3.1.2 Electronic Dynamics Characterization through Electric Dipole Components

**Figure 5:**
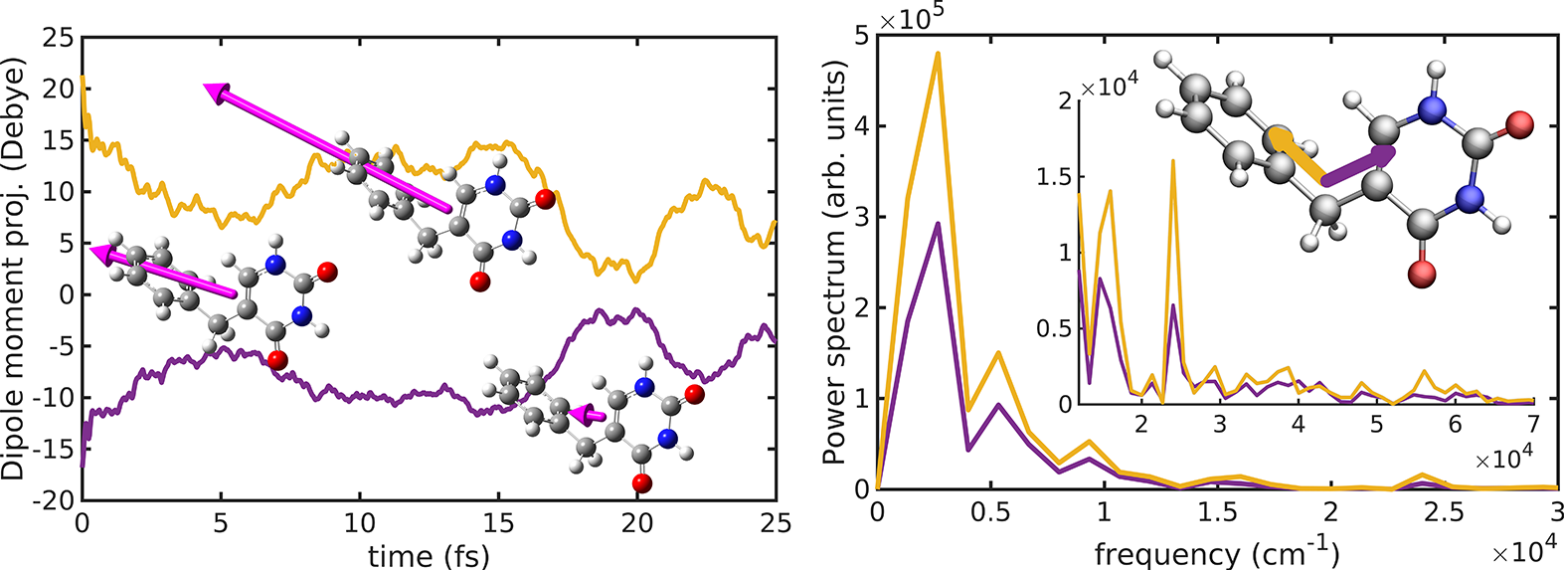
Electric dipole moment projections from 5BU CT electronic dynamics in both time (left panel) and frequency (right panel) domains. Higher frequency contributions are shown in the right panel inset. The bridge-benzene (yellow) and bridge-uracil (purple) projection directions are highlighted. Representative snapshots of the electric dipole moment dynamics are also reported.

The electric dipole moment **μ** is an observable quantity directly linked to the CT state spatial features and the hole/electron relative position. The evolution of **μ** in the 5BU CT electronic dynamics has been projected onto two bridge-benzene and bridge-uracil internal reference directions (Figure 5). Such projections oscillate at positive and negative values, respectively, because of the location of the hole on the benzene moiety and the electron on the uracil one. The electric dipole moment dynamics actually mirrors the relative position of the hole and the electron, which become quite close toward the end (∼20 fs) of the simulation (as demonstrated by a small **μ**). Many frequency contributions (∼9300, 16000, 24000 cm^–1^, up to 56000 cm^–1^) are superimposed on the main low-frequency oscillation, suggesting multiple time-scales for the ultrafast hole/electron rearrangement process.

#### 3.1.3 Electronic Dynamics Characterization through Fragment Charge Cross-Correlations

**Figure 6:**
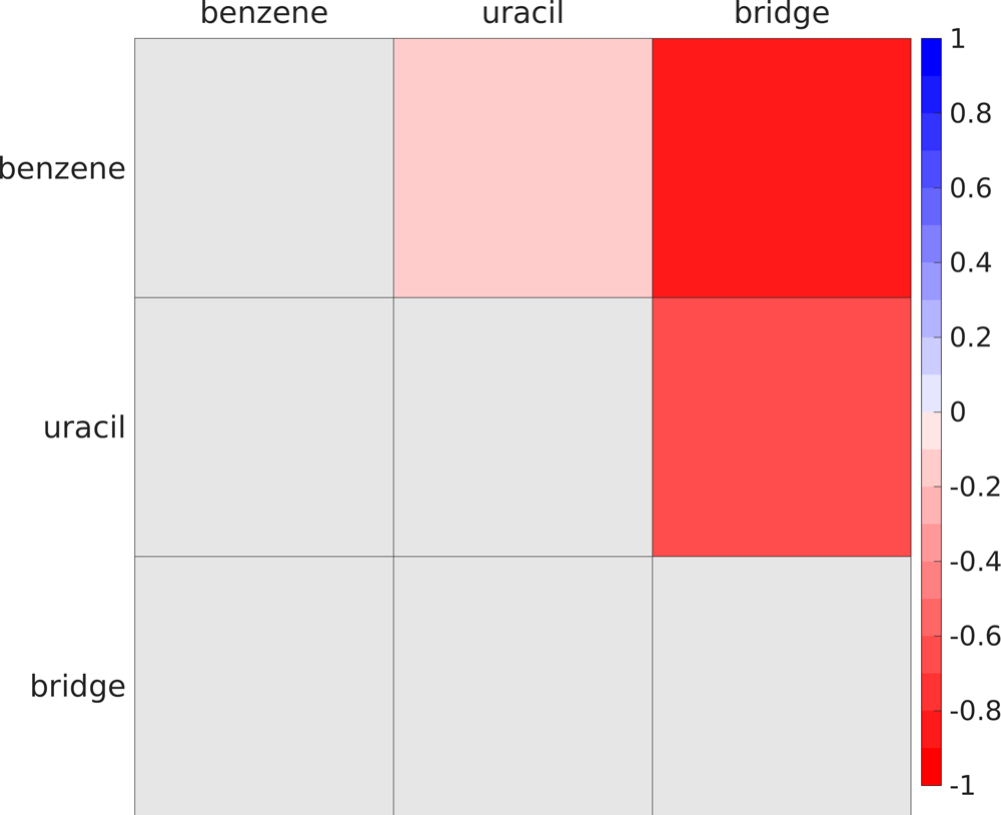
Fragment charges cross-correlation maps from 5BU CT electronic dynamics. Charge cross-correlations for each unique pair of 5BU molecular fragments (Figure 1) are reported. Please refer to Figure 2 for the interpretation and color scale of cross-correlation maps.

In order to characterize the ultrafast 5BU CT electronic dynamics with a higher spatial resolution, a cross-correlation analysis of time-dependent group charges is here introduced besides the previously shown MO occupations and electric dipole moment ones. At the same time, a complete dynamical picture is provided by cross-power spectra, revealing the charge carriers oscillation and transfer frequency. Charge cross-correlation maximum values are collected in cross-correlation maps in order to identify at a glance possible intramolecular charge transfers. Such cross-correlation map (Figure 6) reveals a clear anticorrelated evolution of the time-dependent charges in both the benzene/bridge and uracil/bridge pairs (bright red spots corresponding to high negative cross-correlations, please refer to Table S3 for actual values). This in turn suggests an oscillatory motion of the photoinduced hole and electron which move toward the central CH_2_ bridge moiety. At the same time, the noncorrelation in the benzene/uracil pair reveals that the motion of the two charge carriers is actually not synchronized. The same cross-correlation analysis in the frequency domain (please refer to Figure S8 in the ESI for cross-spectra) shows the very high frequencies (∼250 000 cm^–1^) underlying such anticorrelated motions and therefore the high mobility of both charge carriers.

On average, the evolving hole and electron are ∼1 Å apart around the central bridge (Table 1), while oscillating with a comparable ∼4 Å fs^–1^ mean velocity.

**Table 1:** Hole–Electron RMS Distance  and RMS Velocities ( and ) from 5BU CT RT-TDDFT Dynamics

	*d*_*h*^+^/e^–^_ (Å)	*v*_h^+^_(Å fs^–1^)	*v*_e^–^_(Å fs^–1^)
5BU	1.013	4.263	4.665

### 3.2 N3 Model System

#### 3.2.1 Electronic Dynamics Characterization through MO Occupation Numbers

**Figure 7:**
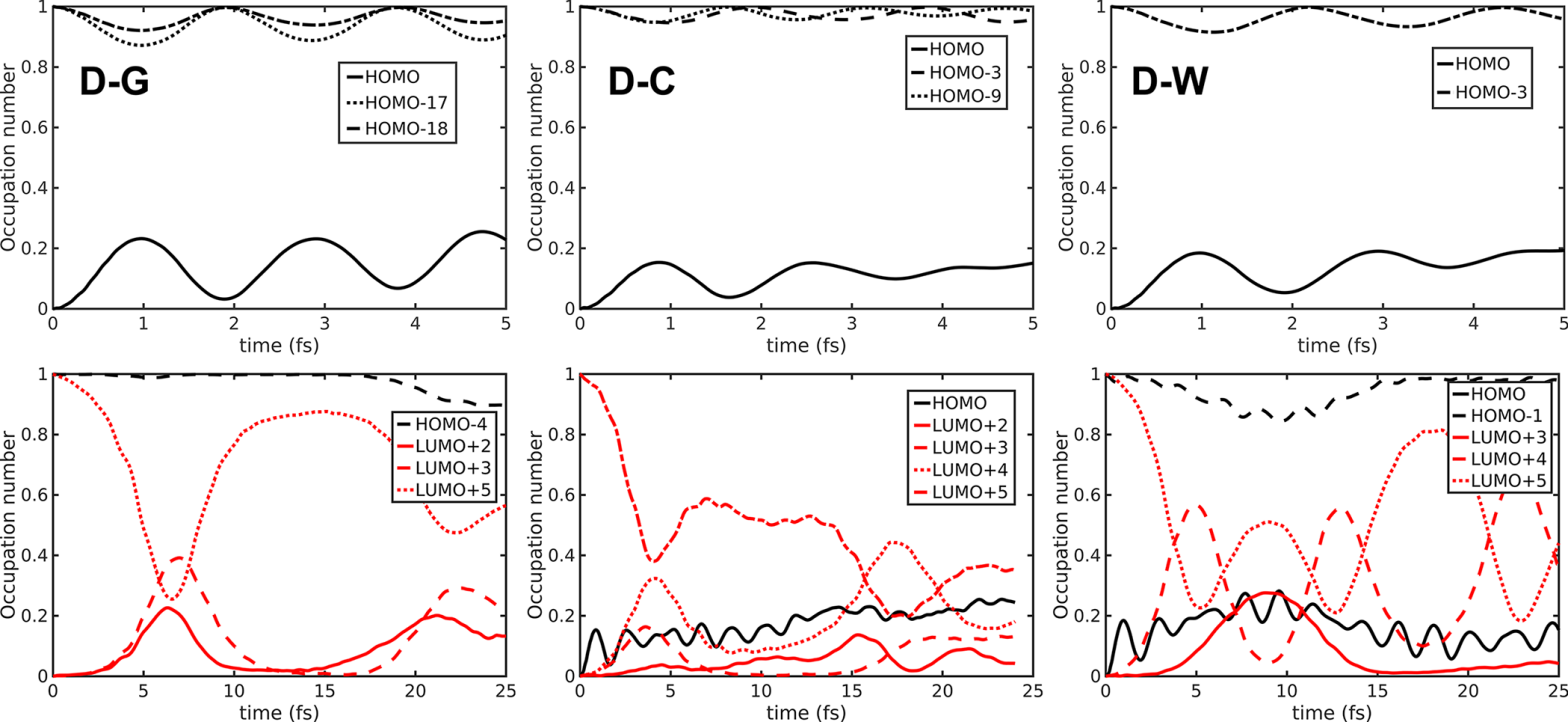
MO occupation number dynamics in N3^4–^^1^MLCT propagation on D-G (left panels), D-C (central panels), and D-W (right panels) structures. Both shorter (5 fs, upper panels) and longer (25 fs, lower panels) time domains are shown to highlight high and low-frequency oscillations, respectively.

Compared to the smaller 5BU model system, the N3^4–^ Ru(II)-complex is a more challenging test case, indeed. In fact, the dynamics of the *n*_*i*_(*t*) MO occupation numbers reveals a complex ultrafast evolution among the dense electronic manifold induced by the ^1^MLCT photoexcitation (Figure 7). Remarkably, a nontrivial occupation dynamics is observed not only for the band-edge orbitals but also for some of the energetically inner MOs. In fact, after the *t* = 0 Koopman excitation (HOMO → LUMO+5, please see Table S2 in the ESI), no sudden hole–electron recombination occurs. The hole created in the HOMO is shared through high-frequency oscillations with inner occupied MOs, all located on the Ru center and on the donor NCS^–^ ligands (HOMO–17 and −18 for D-G, HOMO–3 and −9 for D-C and HOMO–3 for D-W simulations). The photogenerated electron in the LUMO+5 is instead more slowly shared with close unoccupied MOs, mainly centered on the dcbpy acceptor ligands (LUMO+2, +3, and +5 for D-G, LUMO+2, +3, +4, and +5 for D-C and LUMO+3, +4, and +5 for D-W simulations). A substantial loss of LUMO+5 occupation is observed within the first ∼5 fs, but then recovered mainly in D-G and D-W structures simulations.

The MO occupation dynamics allows therefore to give a first qualitative picture about the CT states evolution after photoexcitation. In N3^4–^^1^MLCT in particular, such analysis reveals some features about the different exchange frequency and spatial distribution of the photoinduced hole and electron. Moreover, the treatment of the first shell solvent molecules at a quantum mechanical level (D-W structure) appears to induce wider and more regular oscillations of the occupation of LUMO+5 orbital populated by the photoexcitation, with respect to the simpler, atomic charge based, inclusion of solvent effects (D-C structure). Nevertheless, in systems such as N3^4–^ characterized by closely spaced electronic states, the evolution of MO occupations often appears too involved to give a clear picture, so suggesting the employment of more spatially resolved analysis tools for ED simulations.

#### 3.2.2 Electronic Dynamics Characterization through Electric Dipole Components

**Figure 8:**
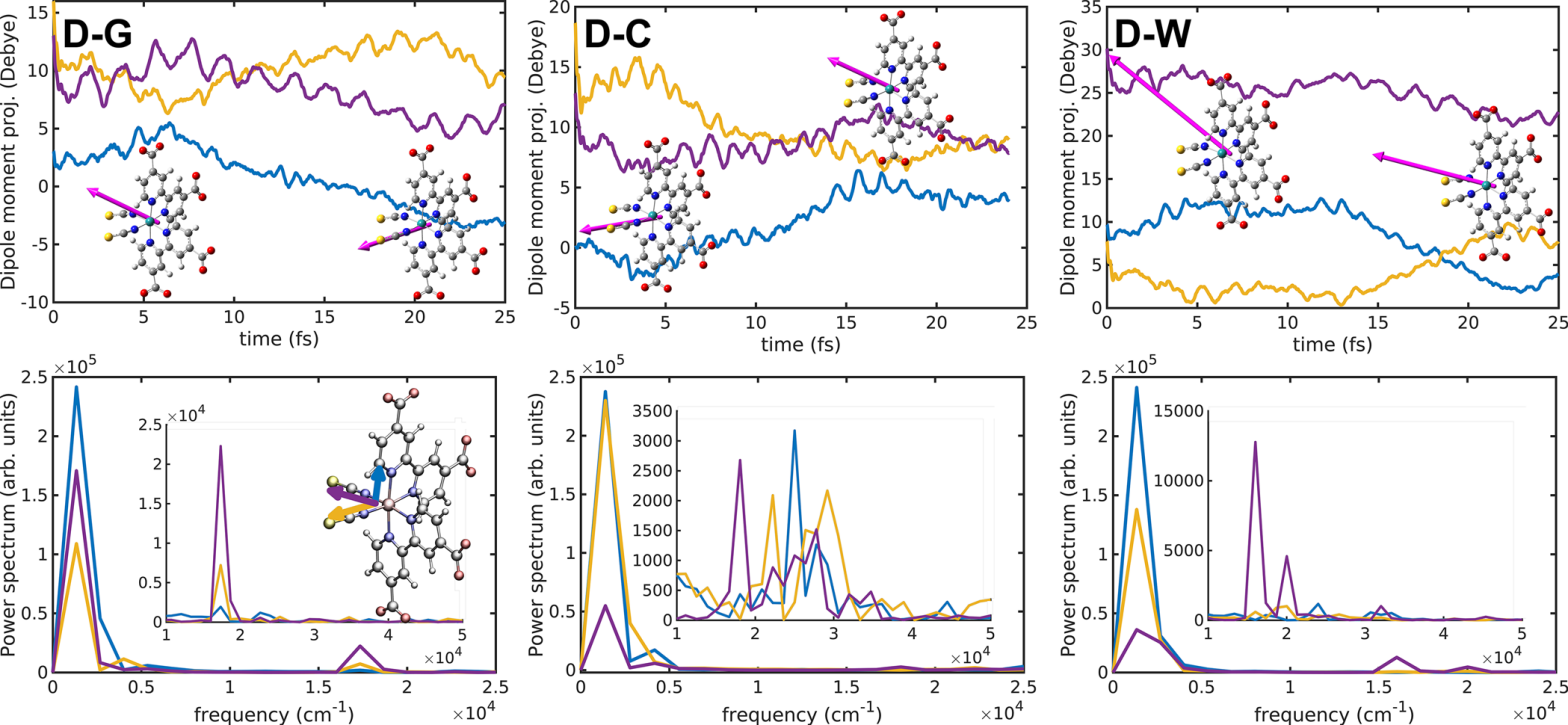
Electric dipole moment projections from D-G, D-C and D-W ^1^MLCT electronic dynamics in both time (upper panels) and frequency (lower panels) domains. The Ru-dcbpy1 (blue), Ru-NCS1 (yellow), and Ru-NCS2 (purple) projection directions are shown in the inset. Representative snapshots of the electric dipole moment dynamics are also reported.

As previously carried out with 5BU CT electronic dynamics, the analysis of the time-dependent electric dipole moment from the N3^4–^^1^MLCT propagation can be simplified by projecting on an internal frame of reference, represented by the (almost) orthogonal Ru-dcbpy1, Ru-NCS1 and Ru-NCS2 directions (see Figure 8 for the definition and color-coding of the projection directions). Therefore, while an axial rotation of **μ** is revealed by a variation of the axial (blue) projection, a rotation in the equatorial plane leads to an anticorrelated motion of the equatorial (yellow and purple) ones. The direction of the electric dipole moment suggests a photoinduced CT from the (NCS)_2_ toward (dcbpy)_2_ sides. In all simulations a high frequency oscillation appears superimposed onto a slower evolution. The former (at ∼17345 cm^–1^ for D-G, 18068 and 25017 cm^–1^ for D-C and 16012 and 20015 cm^–1^ for D-W simulations) are mainly found in the equatorial Ru-NCS1 and Ru-NCS2 projections, likely linked to the fast charge depletion barycenter (hole) motion. The latter instead occurs at a < 1334 cm^–1^ frequency (being this value the resolution resulting from the sampling frequency and the trajectory length) and involves all the dipole moment components. In particular, the concurrent variation of the axial component and the two anticorrelated equatorial ones suggests a helical rotation of the **μ** vector, as also revealed by the snapshots taken at different times (Figure 8, upper panels). This, in turn, is compatible with a migration of the electronic charge accumulation (electron), initially photogenerated on one dcbpy acceptor ligand, toward the other one during the ^1^MLCT propagation. The electric dipole moment dynamics therefore is able to suggest an interligand electron transfer (ILET) event for all the models. CT phenomena in dye sensitizers such as Ru(bpy)_3_^2+^ and N3 have been indirectly investigated through time-resolved spectroscopies, because of their importance for the overall solar cell efficiency, although their ultrafast nature actually prevents any direct experimental observation.^86,123,156−164^

The electric dipole moment analysis in time and frequency domains projected onto specific directions therefore allowed to disentangle the multiple components underlying the MLCT evolution after the photoexcitation. In particular, different frequencies rule the hole oscillation in the Ru(NCS)_2_ moiety and the electron exchange between the dcbpy ligands. The treatment of the solvation as embedded point charges (D-C structure) seems to slightly increase the Ru-NCS components oscillation frequency. A more accurate treatment of the mutual solute–solvent polarization (D-W structure) induces instead a higher dipole moment magnitude and so a higher photoinduced charge separation with respect to the simpler (D-C) or neglected (D-G) modeling of solvent polarization effects.

#### 3.2.3 Electronic Dynamics Characterization through Fragment Charge Cross-Correlations

**Figure 9:**
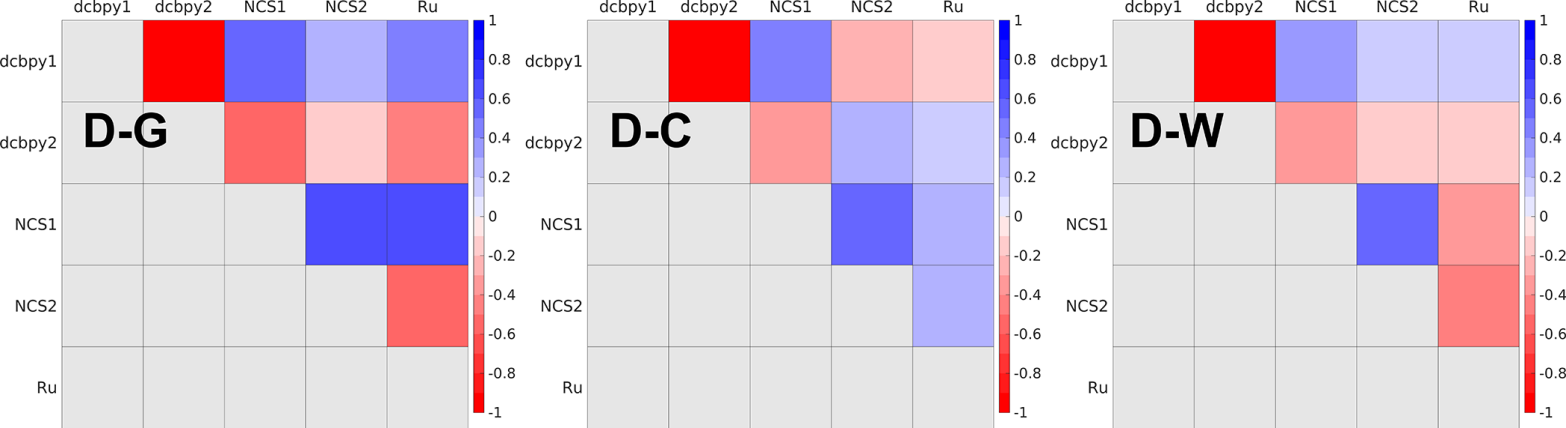
Fragment charges cross-correlation maps from N3^4–^ D-G, D-C, and D-W ^1^MLCT electronic dynamics. Charge cross-correlations for each unique pair of N3^4–^ molecular fragments (Figure 1) are reported.

A spatially resolved analysis of the CT propagation through fragment charges cross-correlations appears even more necessary for the bigger and more complex N3^4–^ model system. In all the N3^4–^^1^MLCT ED simulations a clear electron transfer between the two acceptor dcbpy ligands is apparent (a bright red spot in the cross-correlation maps, Figure 9, corresponding to a ∼−1 anticorrelation; please refer to Tables S4–S6 to actual cross-correlation values and corresponding time-delays). The instantaneous asymmetry of the molecular dye in solution at finite temperature leads in fact to an initial electron photoexcitation mainly on only one dcbpy, followed by its transfer within an ultrafast time-scale. As shown by cross spectra (please see Figure S9 in the ESI), this process always occurs mainly at a low, <1000 cm^–1^, frequency, confirming the results of the previous electric dipole moment analysis, although higher frequency components are also found (D-G: 17345 cm^–1^, D-C: 27797 cm^–1^, D-W: 24017 cm^–1^). NCS^–^ ligands relative charges appear instead always positively correlated to a certain extent (blue spots in the cross-correlation maps), although only in the D-G simulation such correlation occurs without any time-delay (i.e., τ = 0 fs, Table S4). Some charge transfer within the N3^4–^ donor moiety is detected moreover in D-G and D-W simulations, as suggested by moderately anticorrelated (D-G: −0.58, D-W: −0.49 at τ = 0 fs) NCS2 and Ru group charges (red spots in Figure 9). In contrast to the dcbpy electron transfer, only higher frequencies (D-G: 17345, D-W: 16012, and 20015 cm^–1^), comparable to those detected in the electric dipole moment projections, rule this hole exchange.

The partitioning of the time-dependent relative charges on chemically relevant fragments (i.e., the ligands and the metal center) and their time and frequency-domain cross-correlation analysis allowed therefore to highlight ultrafast intramolecular density reorganizations after the photoinduced CT event with a high spatial accuracy. In particular, a lower frequency electron motion between dcbpy ligands and a higher frequency hole exchange between NCS^–^ ligands and the Ru center seem to characterize the ultrafast behavior of N3^4–^ CT states. While the former event is always clearly observed regardless of solvent polarization, a solvent modeling approach through only point charges (D-C structure) seems to (artificially) hide the high-frequency NCS^–^/Ru positive charge exchange, which is instead largely restored with the more accurate QM solvent treatment (D-W structure). A ∼ 1000 cm^–1^ frequency reduction with respect to the gas-phase D-G simulation is moreover induced by solvent polarization.

Average distance and velocities of the charge depletion (hole) and accumulation (electron) also contribute to highlight solvent effects on the ultrafast N3^4–^ CT dynamics (Table 2). In particular, solvation modeling through embedded atomic charges (D-C structure) leads to a contraction of the hole/electron pair, as well as to an increase of their mean velocities, with respect to the gas-phase structure from dynamics (D-G structure). In contrast, the inclusion of mutual solute–solvent polarization at a QM level (D-W structure) actually leads to opposite effects, such as a ∼0.2 Å charge carriers distance increase and a ∼0.4 Å fs^–1^ (i.e., ∼34%) velocity reduction. This further confirms the importance of an accurate treatment of solvation to model the ultrafast electronic dynamics of CT states in solution.

**Table 2:** Hole–Electron RMS Distance  and RMS Velocities ( and ) from N3^4–^^1^MLCT RT-TDDFT Dynamics

	*d*_*h*^+^/e^–^_ (Å)	*v*_*h*^+^_(Å fs^–1^)	*v*_e^–^_(Å fs^–1^)
D-G	2.553	1.266	1.173
D-C	2.196	1.514	1.470
D-W	2.759	0.838	0.760

## 4 Conclusions

The photoinduced CT ultrafast dynamics and excited state electronic properties of two model systems, 5BU and N3^4–^, have been investigated via electronic dynamics simulations.

5BU is a proof-of-concept CT system that still presents a nontrivial electronic dynamics and it is used to mimic photoinduced nucleic acid/protein interactions. The ultrafast CT dynamics of its UV B → U CT state in the gas-phase minimum energy structure shows a quite independent oscillation of the photoinduced hole and electron in the benzene/bridge and uracil/bridge moieties, respectively, also with high (up to ∼250 000 cm^–1^) frequencies. Although no strong correlation between the two charge carriers themselves is detected, our proposed computational and analysis protocol was able to unveil therefore a possible charge recombination mechanism toward the central CH_2_ bridge.

N3^4–^ is instead a popular dye-sensitizer for solar cells and a more challenging model system indeed to test our electronic dynamics analysis protocol. In particular, a photoinduced N3^4–^ MLCT state and the dense electronic manifold implied in the ultrafast relaxation process following the photoexcitation have been computed and analyzed from a dynamical perspective in different environments. RT-TDDFT propagation shows that the photogenerated hole (in the (NCS)_2_/Ru moiety) and electron (on the dcbpy rings) do not appear to recombine in the simulated (25 fs) time, oscillating instead within their respective domains. Very different frequencies seem to characterize the hole (∼16 000 cm^–1^) and the electron (<1000 cm^–1^) oscillations among distinct groups. Simulations carried out on a N3^4–^ structure representative of the dynamics and solvation at finite temperature reveal in particular an interligand electron transfer between the acceptor dcbpy ligands in the ultrafast simulated time regime. The possible effects of a polar solvent onto N3^4–^ excited state electronic manifold dynamics have been then investigated next, where electrostatic and explicit ab initio treatment of solvent molecules have been compared. We have shown that, to achieve an accurate modeling of mutual solute–solvent polarization in N3^4–^ excited states electronic dynamics, first-shell water solvent molecules have to be included at a quantum mechanical level (treating the remaining ones as embedded atomic point charges). In fact, the simpler treatment of solvent as only point charges cannot completely reproduce the dynamics obtained with the more accurate approach. In particular, solvent slows down both charge carriers as can be inspected by computing cross-spectrum frequencies and average velocities, globally reduced with respect to gas-phase ED. When all water molecules are modeled as simpler embedded point charges, the electronic dynamics appears enhanced by such close fixed charges, being however the NCS^–^/Ru hole sharing more faded. The reduced hole–electron distance and higher mean velocities, as well as increased dipole frequencies, are therefore due to an artificially increased polarization effect.

From a methodological perspective, we have shown that RT-TDDFT is here the method of choice to observe the charge density, explicitly propagated in the time domain, along with all time-dependent properties that can be easily extracted from it. A designed protocol of analysis for real-time ED to be applied to time evolving electronic density related properties to characterize both in time and in space CT dynamics in complex systems is here introduced and validated. In our opinion the presented suite of analysis tools to be combined with RT-TDDFT dynamics, performed including large portion of the environment at QM level and describing the resulting part of the system as point charges, can provide direct access to ultrafast charge reorganizations in complex electronic manifolds. As main future applications, the developed techniques can be used to provide a molecular interpretation of the most recent experimental time-resolved spectroscopic techniques, given the subpicosecond nature of the ultrafast charge dynamics processes.

## Supporting Information Available

N3^4–^*ab initio* molecular dynamics in explicit water solution: structural results and computational details; 5BU and N3^4–^ excited states; 5BU and N3^4–^ fragment charges cross-correlations; 5BU and N3^4–^ fragment charges cross-spectra; comparison of population analysis schemes (PDF)
