# Supplementary material for: Understanding Charge Dynamics in Dense Electronic Manifolds in Complex Environments
Source: J Chem Theory Comput. 2023 Jan 5;19(2):626–39. doi: 10.1021/acs.jctc.2c00794 (PMC9878732; doi:10.1021/acs.jctc.2c00794)
Supplement: Supplementary file 1 — ct2c00794_si_001.pdf [file ct2c00794_si_001.pdf]

Electronic Supplementary Information for: Understanding Charge Dynamics in Dense Electronic Manifolds in Complex Environments

Fulvio Perrella,[†,‖] Alessio Petrone,[*,†,‡,¶] and Nadia Rega[*,†,‡,§,¶]

†Department of Chemical Sciences, University of Napoli Federico II, Complesso Universitario di M.S. Angelo, via Cintia 21, I-80126, Napoli, Italy.

‡Scuola Superiore Meridionale, Largo San Marcellino 10, I-80138, Napoli, Italy

¶Istituto Nazionale Di Fisica Nucleare, sezione di Napoli, Complesso Universitario di Monte S. Angelo ed. 6, via Cintia, 80126, Napoli, Italia

§CRIB, Centro Interdipartimentale di Ricerca sui Biomateriali, Piazzale Tecchio 80, I-80125, Napoli, Italy

‖Current address: Scuola Superiore Meridionale, Largo San Marcellino 10, I-80138, Napoli, Italy

E-mail: alessio.petrone@unina.it; nadia.rega@unina.it

1 $N3^{4-}$ *ab initio* molecular dynamics in explicit water solution: structural results and computational details

Computational details

A ground state room temperature ($T = 298\,K$) *ab initio* molecular dynamics (AIMD) of $N3^{4-}$ in explicit water solution was previously collected in Ref. 1.

A spherical box (of 22 Å radius) comprised the $N3^{4-}$ molecule, treated at a QM B3LYP/SDD/def2-SVP level of theory (already validated for this compound[2]) and 1462 water molecules at an MM level, described by the TIP3P force field[3] (Fig. S1). The electrostatic interaction between QM and MM layers was treated including the MM charges in the QM Hamiltonian (i.e., an *electronic embedding*). General AMBER Force Field[4] atom types (and so van der Waals non-bonding parameters) were assigned to the $N3^{4-}$ system. Non-periodic boundary conditions were introduced as a confining potential to avoid solvent diffusion outside the box.[5–8] After a 2 ps equilibration step, a $\sim$8.6 ps production run was collected. A $T = 298\,K$ temperature was kept through velocity rescaling every 1 ps. The Atom-centered Density Matrix Propagation extended Lagrangian approach (ADMP) was employed:[9,10] the density matrix in an orthonormal Gaussian, atom-centered, basis is propagated along with the nuclear degrees of freedom, avoiding a SCF convergence procedure at each step. A mass-weighting scheme which attributes a higher mass to the core functions was chosen, together with a 0.2 amu bohr^2 valence mass. This allowed to employ a 0.1 fs time step.

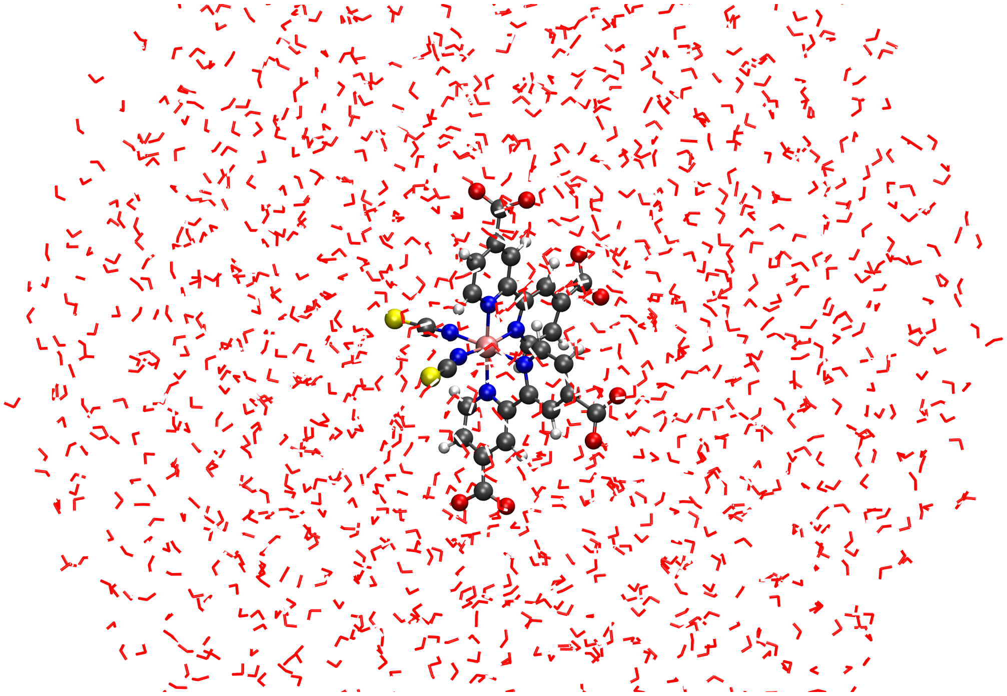

Figure S1: $N3^{4-}$ in a spherical water box. The solute (represented as ball-and-stick) is treated at a QM level, while the surrounding solvent (represented as lines) at a MM level.

Structural results

From the analysis of AIMD in aqueous solution, the $N3^{4-}$ structure is on average distorted with respect to the symmetric C_2-like minimum energy geometry in the gas-phase. Vibrational and environmental effects are indeed able to instantaneously lower such symmetry to some extent. In particular, the isothiocyanate coordination is more bent with respect to the almost linear arrangement of the optimized, gas-phase, symmetric structure ($\sim 167°$ Ru-N(NCS)-C(NCS) angle). The two axial-equatorial N(dcbpy1)-Ru-N(dcbpy2) angles also slightly deviate from the gas-phase value.

Regarding the cybotactic region, dcbpy oxygen and isothiocyanate sulfur atoms are strongly solvated in water solution, interacting with 3-4 solvent molecules in their first solvation shell.

$N3^{4-}$ structural parameters from gas-phase optimized structure and the selected AIMD frame for RT-TDDFT electronic dynamics are compared to AIMD structural distributions.

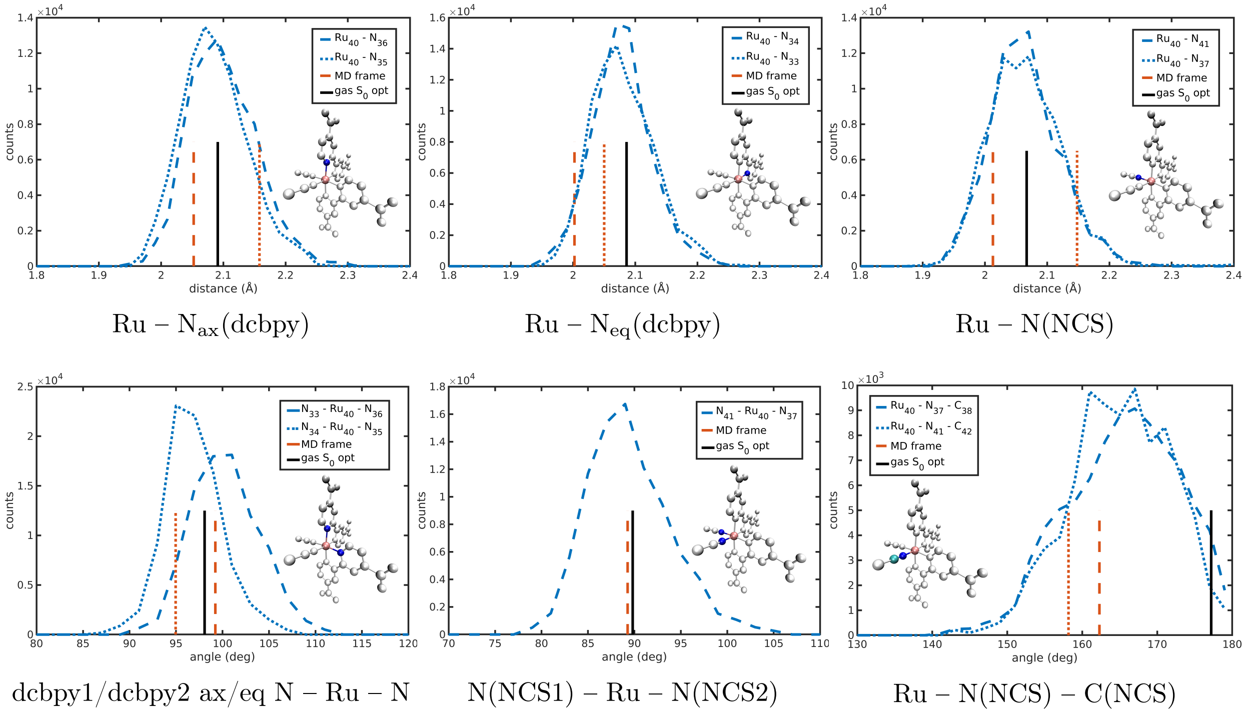

Figure S2: Structural distributions from $N3^{4-}$ *ab initio* molecular dynamics in explicit water solution. Values from an optimized gas-phase structure and the snapshot selected for RT-TDDFT electronic dynamics are shown as vertical black and red bars, respectively.

A continuous symmetry measure (CSM) of $N3^{4-}$ minimal deviation from C_2 symmetry has been evaluated along such trajectory. The index proposed in Refs. 11–13 quantitatively measures the deviation of a structure from its images generated through the symmetry operations of a given point group (C_2 group for $N3^{4-}$), where a resulting lower value in the $[0, 1]$ range corresponds to a more symmetric structure. To improve computational efficiency, a reduced $N3^{4-}$ model (a smaller model able to retain a symmetry not higher than C_2 as the full $N3^{4-}$ structure) has been employed for C_2-CSM calculations. Looking at the distribution from the $N3^{4-}$ AIMD in water solution (Fig. S4), two small CSM values (~ 0.1 and 0.2) appear as the most populated, although higher symmetry distortions (~ 0.45) are occasionally explored. Therefore, compared to the optimized structure (zero CSM value), the dynamical picture offered by the AIMD simulation reveals that $N3^{4-}$ at room temperature in water solution slightly deviates from the C_2 symmetry, due to vibrational motions and solvent fluctuations (mean C_2-CSM: 0.21 ± 0.12). In particular, the $N3^{4-}$ structure from the frame selected for RT-TDDFT electronic dynamics has, in contrast to the optimized, gas-phase, symmetric one, a moderate symmetry distortion (0.13 C_2-CSM value, Fig. S4), belonging to the first highly-populated peak at ~ 0.1.

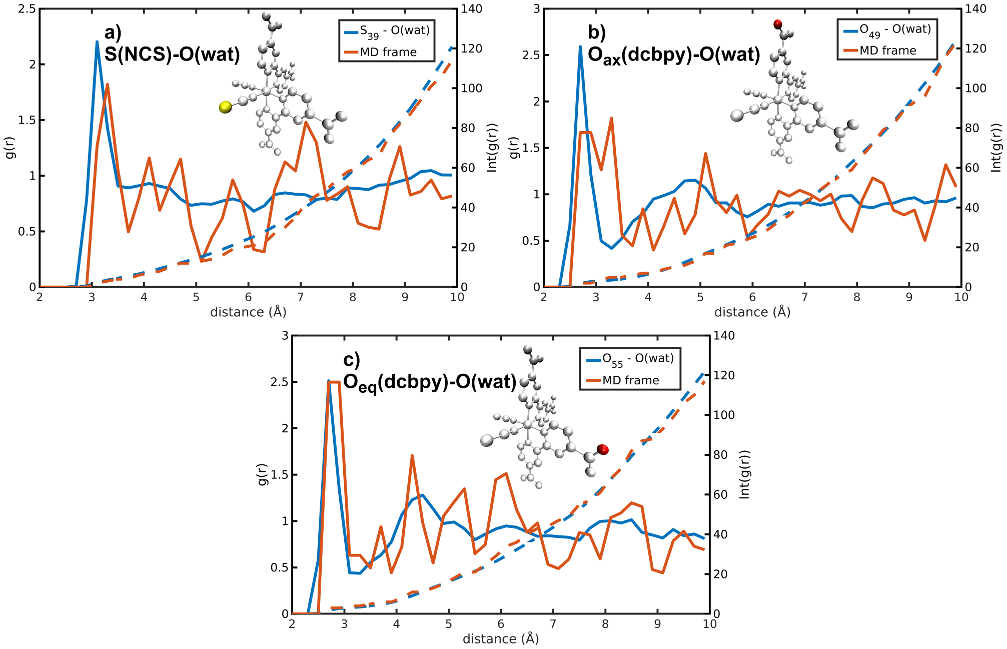

Figure S3: Solute-solvent radial distribution functions (RDF) from *ab initio* molecular dynamics simulation in explicit water solution. Values observed in the AIMD frame selected for RT-TDDFT electronic dynamics are also shown.

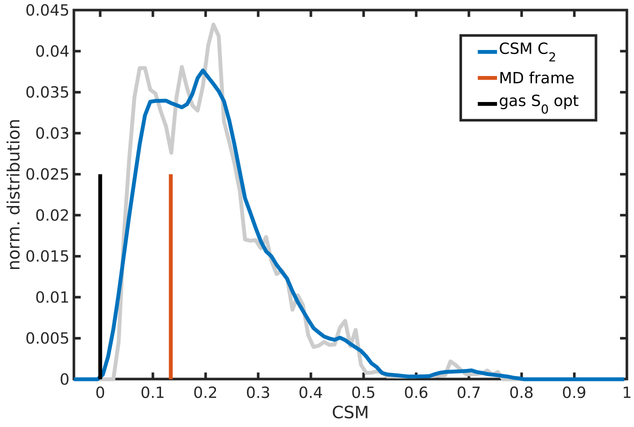

Figure S4: Normalized distribution of the continuous symmetry measure (CSM) with respect to C_2 symmetry group sampled by $N3^{4-}$ AIMD in water solution. CSM values have been calculated with the program provided in Ref. 13. A lower value in the $[0, 1]$ range corresponds to a more symmetric structure. An averaged distribution function (the blue curve) is shown for better clarity. The zero value of the optimized gas-phase $N3^{4-}$ symmetric structure and that (0.13) of the AIMD-derived snapshot selected for MLCT electronic dynamics are also reported as vertical black and red bars (with arbitrary heights), respectively.

2 Excited states

2.1 5BU

Natural Transition Orbitals (NTOs) of the 5BU B → U CT state propagated through RT-TDDFT are shown. NTOs simplify the description of an excited state, providing a couple of hole-electron orbitals.[14] Moreover, a hole-electron correlation plot from transition density population analysis, calculated with TheoDORE software,[15,16] is provided to further characterize the spatial properties of the CT excitation (Fig. S5). The most stable 5BU conformer (with an orthogonal arrangement of the two rings) has been considered.

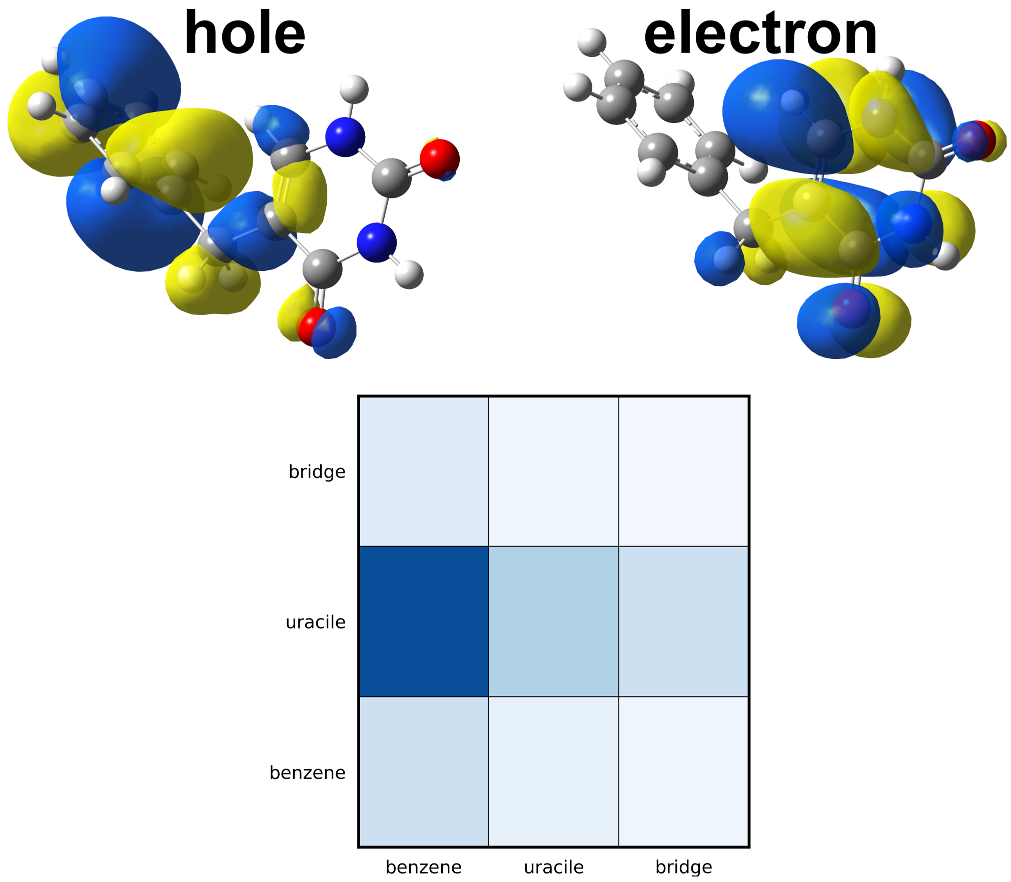

Figure S5: NTOs and hole (horizontal axis)-electron (vertical axis) correlation plot of the 5BU CT state simulated through RT-TDDFT.

Table S1: 5BU CT excited state analysis from LR-TDDFT calculations. In the character column, the MO pairs contributing the most to the corresponding ground to excited singlet state transition (in the parentheses the arriving state) are also reported.

	Energy (cm^{-1})	osc. strength	character
B→U CT	49690	0.0043	HOMO-1 → LUMO (S_6)

2.2 $N3^{4-}$

NTOs (Fig. S6) and hole-electron correlation plots (Fig. S7) of the $N3^{4-}$ ^{1}MLCT state simulated through RT-TDDFT are provided for D-G, D-C and D-W structures.

Table S2: $N3^{4-}$ ^{1}MLCT excited state analysis from LR-TDDFT calculations. In the character column, the MO pairs contributing the most to the corresponding ground to excited singlet state transition (in the parentheses the arriving state) are also reported.

	Energy (cm^{-1})	osc. strength	character	structure
^{1}MLCT	25946	0.0384	HOMO → LUMO+5 (S_{36})	D-G
	27466	0.0724	HOMO → LUMO+5 (S_{14})	D-C
	26441	0.0369	HOMO → LUMO+5 (S_{14})	D-W

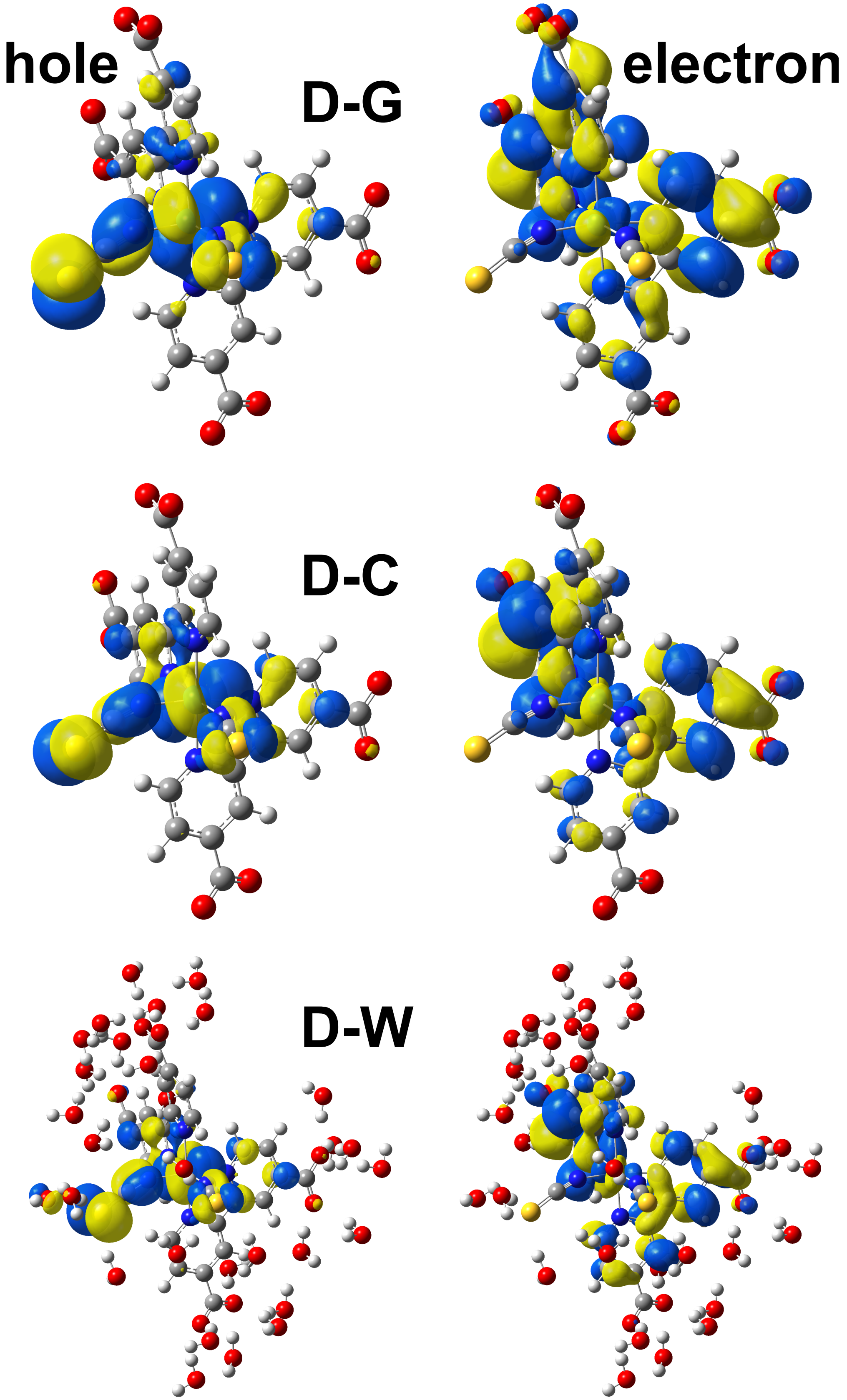

Figure S6: NTOs of the D-G, D-C and D-W $N3^{4-}$ ^{1}MLCT state propagated through RT-TDDFT dynamics.

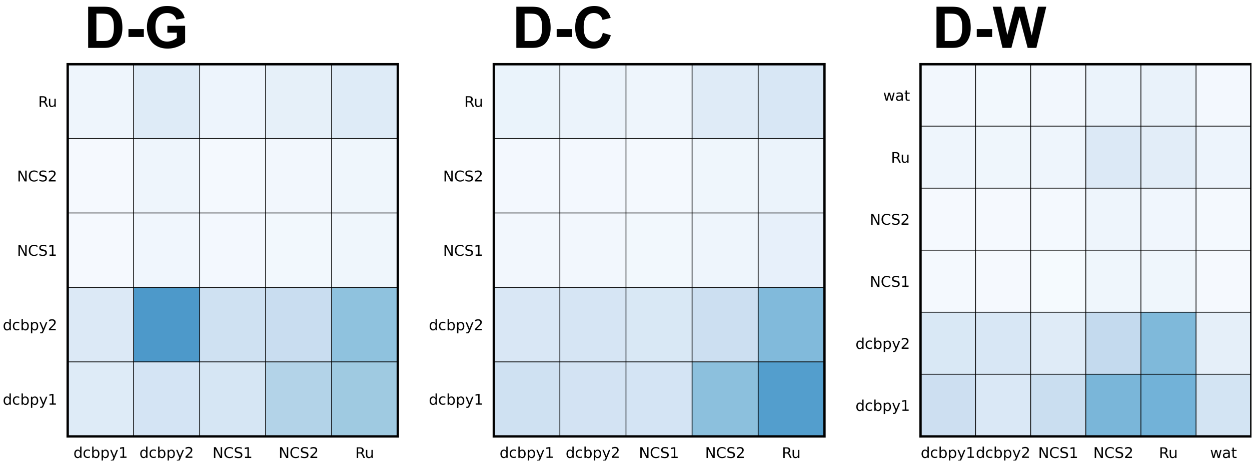

Figure S7: Hole (horizontal axis)-electron (vertical axis) correlation plots of the D-G, D-C and D-W N3^{4-} ^{1}MLCT state propagated through RT-TDDFT dynamics.

3 Fragment charges cross-correlations

The normalized cross-correlations and corresponding time-delays for each unique pair of group charges from 5BU and $N3^{4-}$ excited state electronic dynamics are reported.

3.1 5BU

Table S3: Normalized cross-correlations between fragment charges time series from 5BU CT electronic dynamics. Data are reported as: R_{xy} (τ, fs).

	benzene	uracil	bridge
benzene		-0.20 (0.05)	-0.82 (0.00)
uracil			-0.68 (0.00)
bridge			

3.2 $N3^{4-}$

Table S4: Normalized cross-correlations between fragment charges time series from $N3^{4-}$ ^{1}MLCT electronic dynamics (D-G structure). Data are reported as: R_{xy} (τ, fs).

	dcbpy1	dcbpy2	NCS1	NCS2	Ru
dcbpy1		-0.98 (0.00)	0.54 (0.80)	0.24 (4.70)	0.44 (-1.90)
dcbpy2			-0.57 (-0.05)	-0.19 (4.00)	-0.43 (-0.90)
NCS1				0.66 (0.00)	0.61 (-0.85)
NCS2					-0.58 (0.00)
Ru					

Table S5: Normalized cross-correlations between fragment charges time series from $N3^{4-}$ ^{1}MLCT electronic dynamics (D-C structure). Data are reported as: R_{xy} (τ, fs).

	dcbpy1	dcbpy2	NCS1	NCS2	Ru
dcbpy1		-0.97 (-0.05)	0.41 (-3.70)	-0.28 (3.50)	-0.19 (-0.20)
dcbpy2			-0.38 (-3.60)	0.28 (6.60)	0.17 (5.85)
NCS1				0.52 (1.15)	-0.25 (4.65)
NCS2					0.29 (-0.85)
Ru					

Table S6: Normalized cross-correlations between fragment charges time series from N3^{4-} ^{1}MLCT electronic dynamics (D-W structure). Data are reported as: R_{xy} (τ, fs).

	dcbpy1	dcbpy2	NCS1	NCS2	Ru
dcbpy1		-0.99 (0.00)	0.32 (-0.70)	0.17 (9.90)	0.10 (0.20)
dcbpy2			-0.35 (0.10)	-0.20 (10.40)	-0.11 (0.00)
NCS1				0.53 (3.20)	-0.33 (5.20)
NCS2					-0.49 (0.00)
Ru					

4 Fragment charges cross-spectra

Cross power spectra between selected fragment charges are reported to reveal charge carriers oscillation frequencies.

4.1 5BU

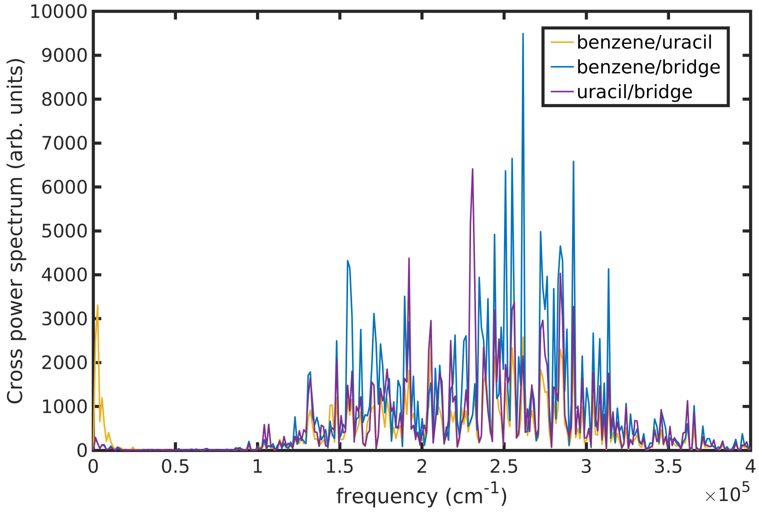

Figure S8: Charge cross power spectra for the three 5BU fragment pairs.

4.2 $N3^{4-}$

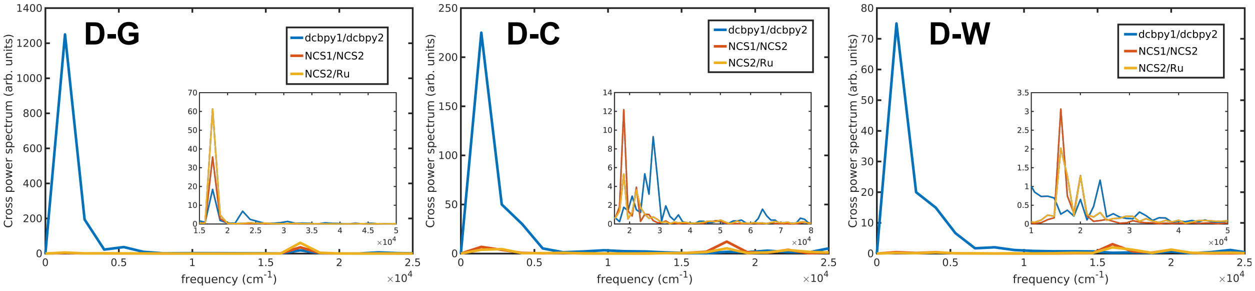

Figure S9: Charge cross power spectra for selected $N3^{4-}$ fragment pairs (D-G, D-C and D-W structures).

5 Comparison of population analysis schemes

Mulliken fragment charges with respect to the ground state at the initial state ($t = 0$) of RT-TDDFT propagation have been compared to the Natural Population Analysis (NPA) ones (Tables S7 and S8).

Table S7: Mulliken and NPA group charges with respect to the ground state at $t = 0$ in the B $\rightarrow$ U CT state RT-TDDFT propagation for 5BU model system.

	Mulliken	NPA
benzene	0.878	0.906
uracil	-0.843	-0.893
bridge	-0.0353	-0.0132

Table S8: Mulliken and NPA group charges with respect to the ground state at $t = 0$ in the MLCT state RT-TDDFT propagation for $N3^{4-}$ model system, D-W structure.

	Mulliken	NPA
dcbpy1	-0.339	-0.336
dcbpy2	-0.490	-0.488
NCS1	0.0548	0.0634
NCS2	0.236	0.252
Ru	0.529	0.501
31 wat	0.00951	0.00729

References

(1) Perrella, F.; Petrone, A.; Rega, N. Direct observation of the solvent organization and nuclear vibrations of $[Ru(dcbpy)_2(NCS)_2]^{4-}$, [dcbpy = (4,4'-dicarboxy-2,2'-bipyridine)], via ab initio molecular dynamics. *Phys. Chem. Chem. Phys.* **2021**, *23*, 22885–22896.

(2) Gaynor, J. D.; Petrone, A.; Li, X.; Khalil, M. Mapping Vibronic Couplings in Intramolecular Charge Transfer of a Solar Cell Dye with Polarization-Selective Two-Dimensional Electronic-Vibrational Spectroscopy. *J. Phys. Chem. Lett.* **2018**, *9*, 6289–6295.

(3) Mark, P.; Nilsson, L. Structure and dynamics of the TIP3P, SPC, and SPC/E water models at 298 K. *J. Phys. Chem. A* **2001**, *105*, 9954–9960.

(4) Wang, J.; Wolf, R. M.; Caldwell, J. W.; Kollman, P. A.; Case, D. A. Development and testing of a general amber force field. *J. Comput. Chem.* **2004**, *25*, 1157–1174.

(5) Brancato, G.; Rega, N.; Barone, V. Molecular dynamics simulations in a NpT ensemble using non-periodic boundary conditions. *Chem. Phys. Lett.* **2009**, *483*, 177–181.

(6) Brancato, G.; Rega, N.; Barone, V. A hybrid explicit/implicit solvation method for first-principle molecular dynamics simulations. *J. Chem. Phys.* **2008**, *128*, 144501.

(7) Rega, N.; Brancato, G.; Barone, V. Non-periodic boundary conditions for ab initio molecular dynamics in condensed phase using localized basis functions. *Chem. Phys. Lett.* **2006**, *422*, 367–371.

(8) Brancato, G.; Barone, V.; Rega, N. Theoretical modeling of spectroscopic properties of molecules in solution: toward an effective dynamical discrete/continuum approach. *Theor. Chem. Acc.* **2007**, *117*, 1001–1015.

(9) Schlegel, H. B.; Millam, J. M.; Iyengar, S. S.; Voth, G. A.; Daniels, A. D.; Scuseria, G. E.; Frisch, M. J. Ab initio molecular dynamics: Propagating the density matrix with Gaussian orbitals. *J. Chem. Phys.* **2001**, *114*, 9758–9763.

(10) Iyengar, S. S.; Schlegel, H. B.; Millam, J. M.; A. Voth, G.; Scuseria, G. E.; Frisch, M. J. Ab initio molecular dynamics: Propagating the density matrix with Gaussian orbitals. II. Generalizations based on mass-weighting, idempotency, energy conservation and choice of initial conditions. *J. Chem. Phys.* **2001**, *115*, 10291–10302.

(11) Zabrodsky, H.; Peleg, S.; Avnir, D. Continuous symmetry measures. *J. Am. Chem. Soc.* **1992**, *114*, 7843–7851.

(12) Pinsky, M.; Casanova, D.; Alemany, P.; Alvarez, S.; Avnir, D.; Dryzun, C.; Kizner, Z.; Sterkin, A. Symmetry operation measures. *J. Comput. Chem.* **2008**, *29*, 190–197.

(13) Pinsky, M.; Dryzun, C.; Casanova, D.; Alemany, P.; Avnir, D. Analytical methods for calculating continuous symmetry measures and the chirality measure. *J. Comput. Chem.* **2008**, *29*, 2712–2721.

(14) Martin, R. L. Natural Transition Orbitals. *J. Chem. Phys.* **2003**, *118*, 4775–4777.

(15) Plasser, F. TheoDORE: A toolbox for a detailed and automated analysis of electronic excited state computations. *J. Chem. Phys.* **2020**, *152*, 084108.

(16) Mai, S.; Plasser, F.; Dorn, J.; Fumanal, M.; Daniel, C.; González, L. Quantitative wave function analysis for excited states of transition metal complexes. *Coordin. Chem. Rev.* **2018**, *361*, 74–97.
